# Cognition enhancing abilities of vitamin D, epalrestat and their combination in diabetic rats with and without scopolamine induced amnesia

**DOI:** 10.1007/s11571-021-09718-6

**Published:** 2021-09-15

**Authors:** Utkarsha D. Kulkarni, Meena Kumari Kamalkishore, Amberkar Mohanbabu Vittalrao, Praveen Kumar Siraganahalli Eshwaraiah

**Affiliations:** Department of Pharmacology, Kasturba Medical College, Manipal, Manipal Academy of Higher Education (MAHE), Manipal, Karnataka 576104 India

**Keywords:** Behavioural assessment, Cognitive impairment, Diabetes mellitus, Glutathione malondialdehyde, Morris water maze, Passive avoidance

## Abstract

Persistent hyperglycaemia and scopolamine were used to inflict amnesia in rats. Chronic hyperglycaemia causes metabolic impairment, neuronal dysfunction and oxidative stress causing cognitive impairment. This study aimed to determine anti amnesic activities of vitamin D, epalrestat and their combination against diabetes and scopolamine induced cognitive dysfunction. A total of eighty-eight *Wistar albino rats*, eleven groups, and 8 rats/Gr., were used. Type 2 diabetes mellitus was induced in all groups, except Gr.1 which was treated with 2 ml normal saline. Gr. 2 to 11 by feeding high fat diet for 28 days followed by single dose streptozotocin 35 mg/kg i.p. Hyperglycemic rats were screened with blood sugar level > 200 mg/dL. Gr. 2 rats were treated with only streptozotocin and Gr. 3 to 6 were treated with streptozotocin and test drugs donepezil 1 mg/kg, vitamin D, 27 mcg/kg, epalrestat 57 mg/kg, vitamin D + epalrestat, per oral, respectively. Gr. 7 rats were treated with only streptozotocin + scopolamine and all others from Gr. 8 to 11 were treated with streptozotocin + scopolamine and donepezil, vitamin D, epalrestat, vitamin D + epalrestat respectively. The gold standard behavioural tests were conducted by using Morris water maze and passive avoidance paradigms after 30–60 min of inj. scopolamine, 0.5 mg/kg, intra-peritoneal. Hippocampal tissue was taken for histopathological and biochemical evaluation. Rats treated with donepezil, vitamin D, epalrestat and vitamin D + epalrestat showed significant improvement in behavioural, biochemical and histopathological parameters as compared to streptozotocin and (streptozotocin + scopolamine) treated rats. This study underscores cognition enhancing abilities of vitamin D and epalrestat, and their combination in diabetic rats with and without scopolamine.

## Introduction

Diabetes Mellitus (DM) is a dynamic metabolic disease that can have debilitating effects on numerous organ systems in the body, including the nervous system, through a variety of mechanisms (Kodl and Seaquist 2008). One of the well-recognized but less addressed complications of diabetes is cognitive dysfunction leading to impairment of learning and memory. Period of cognitive dysfunction in DM is thought to be linked to increased apoptosis and reduced neuronal density in hippocampus diabetic rats (Li et al. 2002). Factors which play possible important role in pathogenesis of diabetes induced learning and memory deficit are (I) oxidative stress, (II) metabolic impairment in the form of diabetic hyperglycaemia due to insulin resistance and (III) vascular complications (Hasanein and Shahidi 2010). Oxidative stress is generally recognized as the primary mediator for the production and progression of diabetic complications. Oxidative stress is characterized as a chain of events caused by an imbalance between oxidative substances and antioxidant ability, followed by an exacerbated development of reactive oxygen species and weakened antioxidant defences (Li et al. 2016). The hippocampus and the amygdala are important in the consolidation of learning and memory. Hippocampus is mainly involved in consolidation of declarative memory (Halbach 2007). Short-term memory conversion into long-term memory is severely affected when hippocampus is damaged. Spatial memory is also affected in case of hippocampal damage (Burgess et al. 2001).

Diabetic hyperglycaemia can cause a polyol pathway that converts glucose to sorbitol by a rate-limiting enzyme called aldose reductase. Sorbitol can be converted to fructose using fructose reductase, but the absence of fructose kinase in peripheral nervous tissue causes sorbitol to build up intracellular hypertonia and inhibits inositol uptake (Kodl and Seaquist 2008). Intracellular inositol is depleted, resulting in oedema, demyelination and necrosis of nerve cells. These mechanisms may be operational in the brain and may contribute to alterations in cognitive function that have been identified in patients with diabetes (Kodl and Seaquist 2008). Epalrestat (5-[(1*Z*, 2*E*)-2-methyl-3-phenyl propenylidene]-4-oxo-2-thioxo-3-thiazolidine acetic acid) is an aldose reductase inhibitor (ARI) authorized in Japan for the treatment of diabetic neuropathy and considered to have limited adverse effects with the well-proven antioxidant and anti-inflammatory properties (Hotta et al. 2006). Anti-inflammatory and antioxidant effects are recorded with rat Schwann cell and human neuroblastoma cell line (Yama et al. 2016; Sato et al. 2013; Ohmura et al. 2009). 1, alpha-25 dihydroxyvitamin D3 (1, 25-(OH)_2_ D_3_) is associated with the development and functioning of the brain (Mathieu and Badenhoop 2005; Garcion et al. 2002). Some vitamin D target gene products and their regulated processes are known to be involved in the critical functions necessary for cognition and behaviour. Vitamin D can also be involved in the planning, processing and formation of learning and memory (Garcion et al. 2002; McCann and Ames 2008; Moghadamnia et al. 2015; Buell et al. 2009). Thus both vitamin D and epalrestat have found to have the positive effect on learning and memory, therefore this study aims to combine vitamin D and epalrestat to assess the effect of this combination and compare it with donepezil which is a cerebro-selective inhibitor of acetyl-cholinesterase (AChE).

## Materials and methods

### Animals

Eighty-eight, male, adult (8–10 weeks old) Albino Wistar rats (*Rattus norvegicus*) weighing 150–200 g were used for this study. Animals were taken from the Central Animal Research Facility of Manipal Academy of Higher Education, Manipal. Animals were maintained at temperature 28 °C, humidity approximately 50 ± 10% and 12:12 light–dark cycle. All the control group rats received standard animal feed (VRK Nutritional Solutions, Pune, India) and rats belonging to test groups received high fat diet (HFD). The animals were housed in each polypropylene cage of size 41 cm × 28 cm × 14 cm consisting of sterile paddy husk bedding which was changed every alternative day. Institutional Animal Ethical Committee clearance was obtained before the start of the study.

### Experimental induction of diabetes

Type-2 diabetes was induced in test groups by feeding rats with high fat diet for 4 weeks (Guo et al. 2012; Zhang et al. 2008) followed by low dose (35 mg/kg) intraperitoneal injection of streptozotocin (STZ). STZ treated rats received 5% of glucose instead of water for 24 h after STZ injection in order to reduce hypoglycaemic shock related mortality (Jaiswal et al. 2018a). Blood samples were collected from the tail vein 48 h after STZ to measure glucose levels. Animals with fasting blood glucose levels above 200 mg/dL were considered diabetic and used for further study (Srinivasan et al. 2005). Excessive urination was an additional indicator of diabetes. The composition of HFD was as given in Table 1 (Srinivasan et al. 2005).

**Table 1:** Composition of high fat diet

Ingredients	g/kg
Powdered normal pellet diet (NPD)	365
Lard	310
Casein	250
Cholesterol	10
Vitamin and mineral mix	60
DL-methionine	03
Yeast powder	01
Sodium chloride	01

Drugs/chemicals/instruments:

Following drugs and testing kits were used in the study:

Drugs: Streptozotocin (Everon Life Sciences, New Delhi), Vitamin D (Elcon’s Vitamin D_3_, 5000 IU, Elcon Drugs and Formulations Ltd.), Epalrestat (Eparel, 50 mg, Micro Labs Ltd.), Donepezil (Alzil 5 mg, Intas Pharmaceutical Ltd.), Scopolamine (Sigma-Aldrich, Bangalore).

Lab. Kits used: (1) MDA (Lipid Peroxidation (MDA) Assay Kit sufficient for 100 colorimetric or fluorometric tests), (2) GSH (Reduced Glutathione (GSH) Assay Kit (Colorimetric) sufficient for 100 colorimetric tests) from Sigma-Aldrich Merck, Bangalore and (3) AChE kits (Acetylcholinesterase Fluorescent Activity Kit) from Invitrogen Bioservices India Pvt Ltd, Bangalore.

Ingredients required to prepare HFD were purchased from Shree distributors, Udupi. All the reagents and instruments required for biochemical and histopathological analysis were obtained from the departmental pharmacology lab, including the instruments needed for dissection (Table 2).

**Table 2:** Experimental groups

Groups	Drug administered	Dosage and route
1	Control	2 ml NS, orally
2	STZ	35 mg/kg, i.p
3	STZ + donepezil	35 mg/kg, i.p + 1 mg/kg, orally
4	STZ + vitamin D	35 mg/kg, i.p + 27mcg/kg, orally
5	STZ + epalrestat	35 mg/kg, i.p + 57 mg/kg, orally
6	STZ + (vitamin D + epalrestat)	35 mg/kg, i.p + (27mcg/kg, orally + 57 mg/kg, orally)
7	STZ + scopolamine	35 mg/kg, i.p + 0.5 mg/kg, i.p
8	(STZ + scopolamine) + donepezil	(35 mg/kg, i.p + 0.5 mg/kg, i.p) + 1 mg/kg, orally
9	(STZ + scopolamine) + vitamin D	(35 mg/kg, i.p + 0.5 mg/kg, i.p) + 27mcg/kg, orally
10	(STZ + scopolamine) + epalrestat	(35 mg/kg, i.p + 0.5 mg/kg, i.p) + 57 mg/kg, orally
11	(STZ + scopolamine) + (vitamin D and epalrestat)	(35 mg/kg, i.p + 0.5 mg/kg, i.p) + (27mcg/kg, orally + 57 mg/kg, orally)

*Administration of STZ* STZ (35 mg/kg) dissolved in 0.05 M chilled Na citrate buffer with pH of 4.5 was given as a single dose i.p injection (Jaiswal et al. 2018a).

*Administration of scopolamine* Scopolamine 0.5 mg/kg i.p 30 min prior to behavioural studies (Sumanth et al. 2010).

*Administration of vitamin D* Vitamin D 27mcg/ kg daily given per oral for 4 weeks. Vitamin D dissolved in 0.3 ml Tween® 80 (Mathieu and Badenhoop 2005).

*Administration of epalrestat* Epalrestat 57 mg/kg per oral for 4 weeks (Jaiswal et al. 2018b).

*Administration of donepezil* Donepezil 1 mg/kg/day per oral for 4 weeks (Jaiswal et al. 2018a).

### Behavioural assessment

#### Morris water maze

Water maze experiment was carried out described by *Morris R* (Morris 1984). The apparatus consists of a round tank (165 cm × 35 cm) the tank was filled with water and maintained at 250 °C. Powdered milk was added to water to remove transparency. The apparatus was divided into 4 equal zones (NE, NW, SE, and SW). A platform (10 cm^2^) was kept in one of the zones just submerged in water. Black and white symbol board was placed as cue in a target zone. The position of extra maze cue and platform were kept constant throughout the learning sessions. The water maze test consists of two phases (Vorhees and Williams 2006).

- Spatial task acquisition phase

Four trials (2 min) are done for every animal for 4 days continuously during which the study animal was trained to escape from cold water by reaching hidden platform and staying there for 20 s. Four start positions were used (North, South, East, and West). Each day animals were given a series of daily trials with random start positions. In the event that the rat was unable to locate the platform even after 90 s, it was manually directed to the platform. Time to reach the platform was counted. A preliminary study was undertaken to acquaint the rat with the water maze.

(b) Probe trial

On the last day of the experiment, the platform was taken away. The animal was kept in a new place in the maze, directed towards the tank wall in opposite quadrant as that of the original target quadrant. The animal was removed after 30 s and time and distance travelled in the target zone was measured.

#### Passive avoidance (PA) test

- Apparatus

Behavioural assessment of learning and memory was done using PA test (Trnečková et al. 2005). The setup comprises of two compartments, namely an illuminated one and a dark. There is an opaque guillotine door between these compartments. The floor of the two compartments is made of stainless steel rods, but the floor of the dark part could be wired with electrical current.

(b) Training

The rat was placed, in the illuminated compartment, facing away from the guillotine door, and 5 s later, the door was opened. When the rat went into the dark compartment, the door was shut and a 50 Hz square wave, 1 mA constant current shock was applied for 1 s. The rat was kept in the apparatus and received a foot shock every time it entered the dark compartment. The acquisition was stopped when the rat stayed in the illuminated compartment for 90 consecutive seconds.

(c) Retention

During the retention test, which was conducted 24 h later than acquisition test, every rat was again placed in the illuminated compartment. The interval between placement in the illuminated compartment and entry into the dark compartment was computed as step through latency (STL). The STL and the time spent in the dark compartment were recorded as an indicator of retention performance. The maximum time for which an animal was made to stay in the apparatus or ceiling time was considered as 180 s and behavioural tests was performed at 8–12 h.

After behavioural assessment, animals were sacrificed by injecting lethal dose of phenobarbitone sodium and whole body was perfused initially with heparinized saline and then with 10% formalin using trans-cardiac perfusion technique. Then the brain was carefully dissected out and placed in ice cold phosphate buffered saline (0.1 M, pH 7.4) for biochemical analysis. For histopathology, brain tissues were fixed in neutral buffered formalin (10%).

### Biochemical analysis

#### Malondialdehyde (MDA) estimation

Lipid peroxidation was estimated by measuring MDA levels in brain as per Okhawa et al. method (Ohkawa et al. 1979; Satoh 1978). MDA levels in brain homogenate were determined by using thiobarbituric acid (TBA), which gives a red coloured compound exhibiting peak absorbance at 532 nm which was measured by spectrophotometer.

#### Glutathione (GSH) estimation

Glutathione was estimated by using Elman’s protocol (Ashwlayan and Singh 2017). Elman’s reagent (5,5′-dithiobis-(2-nitrobenzoic acid) or DTNB) is reduced by –SH groups present in GSH to give yellow coloured compound exhibiting peak absorbance at 412 nm which can be measured by spectrophotometer.

#### Acetylcholine esterase (AChE) estimation

AChE activity was quantitatively measured by Elman’s method (Ellman et al. 1961). Elman’s reagent (DTNB) reacts with thiocholine to give a compound which exhibits yellow colour and shows absorbance at 412 nm. The enzyme activity was determined by recording the rate of change in absorbance at 412 nm by using spectrophotometer.

### Statistical analysis

Graph Pad Prism 5.0 software was used for analysing the data. Results were analysed by using one-way analysis of variance (ANOVA), followed by post hoc Tukey’s test. The level of statistical significance for any measure was set at *p* < 0.05 at a confidence interval of 95%. The data is expressed as mean ± standard deviation.

## Results

### Behavioural assessment

Morris water maze and passive avoidance test were conducted and the findings were analysed.

#### Morris water maze

**Fig. 1:**
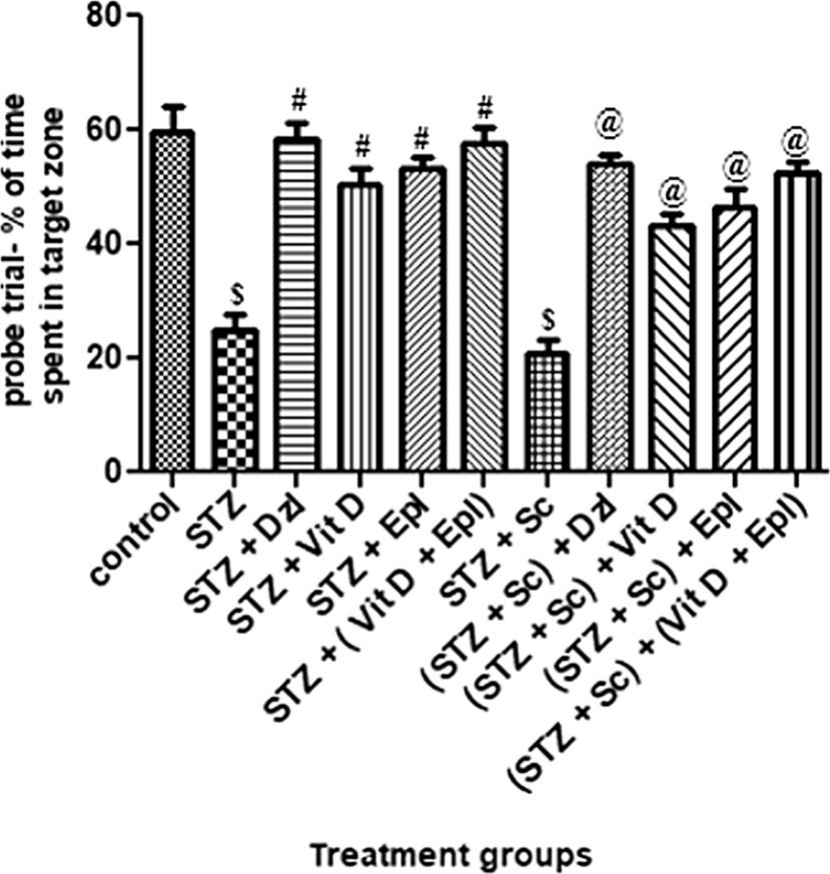
Effect of various drugs on % of time spent in target zone in probe trial of MWM. STZ: Streptozotocin, Sc: Scopolamine, Epl: Epalrestat, Vit D: Vitamin D, Dzl: Donepezil. ^$^*p* < 0.001 versus control, ^#^*p* < 0.05 versus STZ, ^@^*p* < 0.05 versus [STZ + Sc]. Values are expressed as Mean ± SD. Comparison is done by one-way ANOVA followed by post hoc Tukey’s analysis

**Fig. 2:**
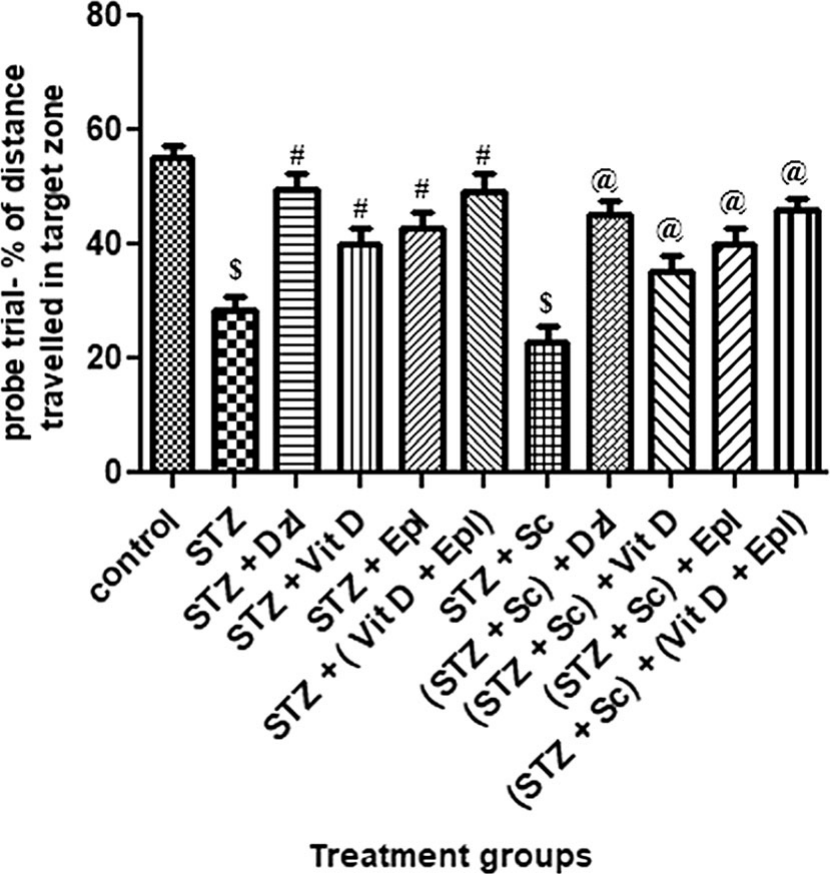
Effect of various drugs on % of distance travelled in target zone in probe trial of MWM. STZ: Streptozotocin, Sc: Scopolamine, Epl: Epalrestat, Vit D: Vitamin D, Dzl: Donepezil. ^$^*p* < 0.001 versus control, ^#^*p* < 0.05 versus STZ, ^@^*p* < 0.05 versus [STZ + Sc]. Values are expressed as Mean ± SD. Comparison is done by one-way ANOVA followed by post hoc Tukey’s analysis

*Probe trial: Percentage of time spent and distance travelled in target zone* In reference to Figs. 1 and 2, in probe trial conducted on day 5, the percentage of time spent and percentage of distance travelled in target zone by rats treated with STZ and (STZ + scopolamine) decreased significantly (*p* < 0.001) as compared to control rats.

It was observed that the percentage of time spent and percentage of distance travelled in target zone by groups treated with donepezil, vitamin D, epalrestat and (vitamin D + epalrestat) increased significantly (*p* < 0.05) as compared to the STZ group. However, percentage of time spent and percentage of distance travelled in target zone by donepezil group was comparable with that of (vitamin D + epalrestat) group.

In scopolamine treated set of rats, there was a significant (*p* < 0.05) increase in percentage of time spent and percentage of distance travelled in target zone by the groups dosed with donepezil, vitamin D, epalrestat and (vitamin D + epalrestat) as compared to (STZ + scopolamine) group. Percentage of time spent and percentage of distance travelled in target zone by donepezil group was comparable with that of (vitamin D + epalrestat) group. (Figs. 1, 2).

#### Passive avoidance test

- Step through latency (STL)

**Fig. 3:**
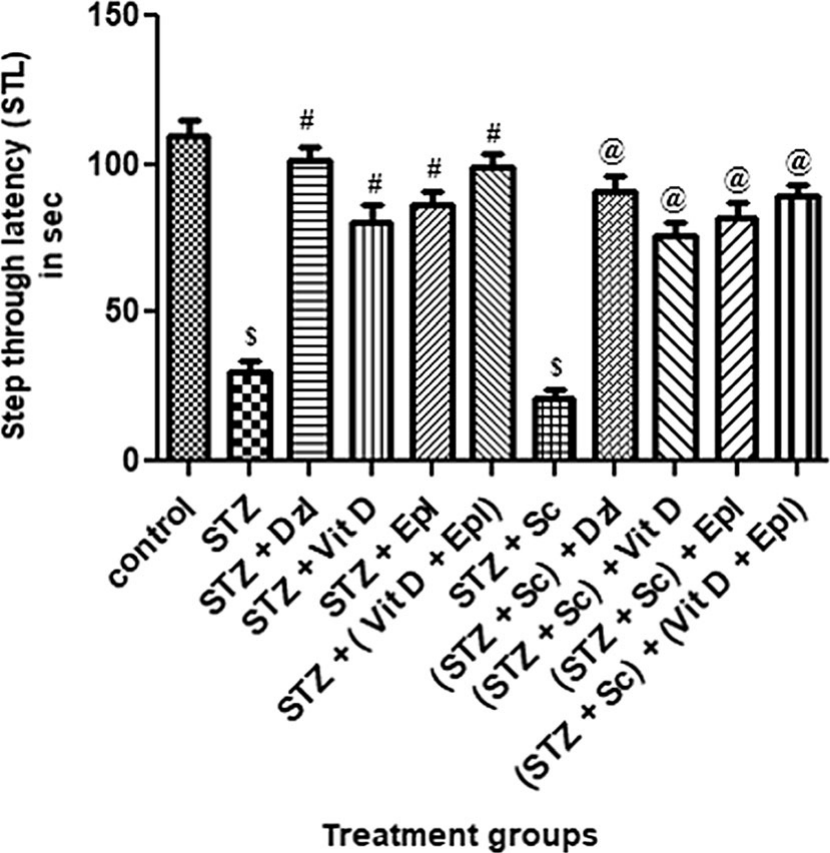
Effect of various drugs on step through latency (STL) in passive avoidance test. STZ: Streptozotocin, Sc: Scopolamine, Epl: Epalrestat, Vit D: Vitamin D, Dzl: Donepezil. ^$^*p* < 0.001 versus control, ^#^*p* < 0.05 versus STZ, ^@^*p* < 0.05 versus [STZ + Sc]. Values are expressed as Mean ± SD. Comparison is done by one-way ANOVA followed by post hoc Tukey’s analysis

As depicted in Fig. 3, for retention trial, step through latency (STL) or time taken by the animal to enter the dark compartment was measured. There was a significant (*p* < 0.001) reduction in STL of rats treated with STZ and (STZ + scopolamine) as compared to control rats.

In comparison with STZ group, there was a significant (*p* < 0.05) increase in the STL for groups treated with donepezil, vitamin D, epalrestat and (vitamin D + epalrestat). Step through latencies of donepezil group was comparable with STL of (vitamin D + epalrestat) group.

Among scopolamine treated groups, STL of groups treated with donepezil, vitamin D, epalrestat and (vitamin D + epalrestat), increased significantly (*p* < 0.05) than that of the group treated with (STZ + scopolamine). Step through latencies of donepezil group was comparable with STL of (vitamin D + epalrestat) group (Fig. 3).

(b) Time spent in the dark compartment

**Fig. 4:**
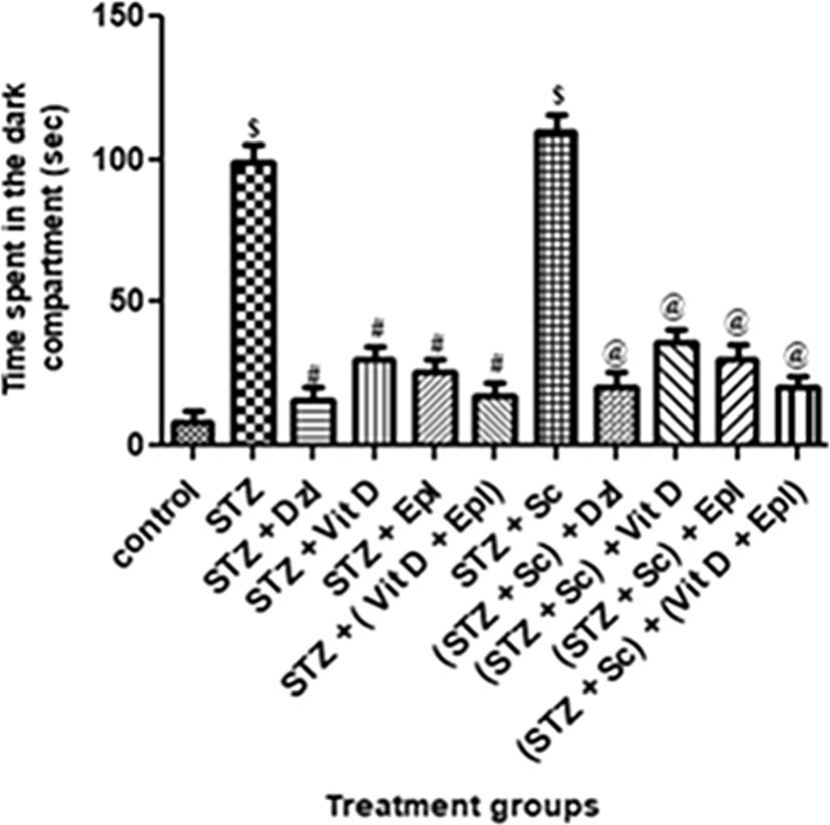
Effect of various drugs on time spent in the dark compartment in passive avoidance. STZ: Streptozotocin, Sc: Scopolamine, Epl: Epalrestat, Vit D: Vitamin D, Dzl: Donepezil. ^$^*p* < 0.001 versus control, ^#^*p* < 0.05 versus STZ, ^@^*p* < 0.05 versus [STZ + Sc]. Values are expressed as Mean ± SD. Comparison is done by one-way ANOVA followed by post hoc Tukey’s analysis

As seen in Fig. 4, time spent in the dark compartment increased significantly (*p* < 0.001) for groups treated with STZ and (STZ + scopolamine) as compared to control group.

Groups treated with donepezil, vitamin D, epalrestat and (vitamin D + epalrestat), showed significant (*p* < 0.05) decrease in time spent in the dark compartment as compared to STZ group. But time spent in the dark compartment by donepezil group was comparable with that of (vitamin D + epalrestat) group.

Similarly, for scopolamine treated set of rats, groups dosed with donepezil, vitamin D, epalrestat and (vitamin D + epalrestat), showed significant (*p* < 0.05) decrease in time spent in the dark compartment as compared to (STZ + scopolamine) group. However, time spent in the dark compartment by donepezil group was comparable with that of (vitamin D + epalrestat) group (Fig. 3).

### Biochemical analysis

Malondialdehyde (MDA), glutathione (GSH) and acetyl cholinesterase (AChE) were measured in brain homogenate.

#### Malondialdehyde (MDA) analysis in brain homogenate

**Fig. 5:**
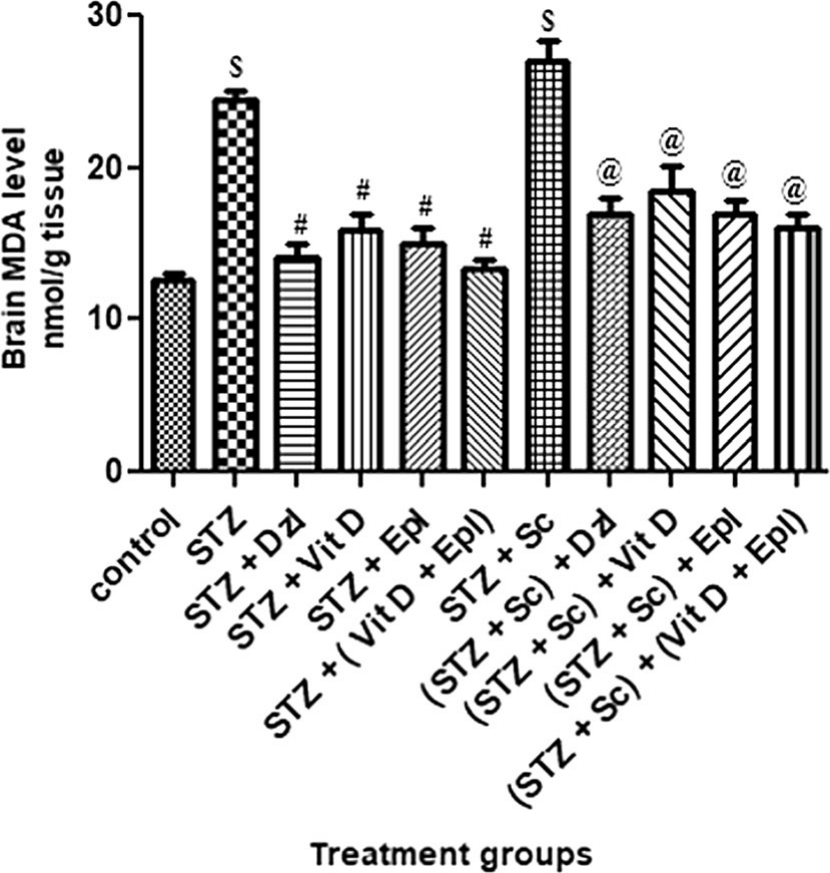
Effect of various drugs on MDA levels in brain homogenate. STZ: Streptozotocin, Sc: Scopolamine, Epl: Epalrestat, Vit D: Vitamin D, Dzl: Donepezil. ^$^*p* < 0.001 versus control, ^#^*p* < 0.05 versus STZ, ^@^*p* < 0.05 versus [STZ + Sc]. Values are expressed as Mean ± SD. Comparison is done by one-way ANOVA followed by post hoc Tukey’s analysis

As reflected in Fig. 5, rats treated with STZ and (STZ + scopolamine) showed significant (*p* < 0.001) increase in brain MDA levels as compared to control rats. Rats treated with donepezil, vitamin D, epalrestat and (vitamin D + epalrestat), MDA levels were significantly (*p* < 0.05) reduced as compared to STZ and (STZ + scopolamine) treated group of rats. However, the combination (vitamin D + epalrestat) treated group of rats statistically showed significant reduction in the MDA levels in diabetic rats with and without the scopolamine drug; which were comparable with that of donepezil treated rats (Fig. 5).

#### Glutathione (GSH) levels in brain homogenate

**Fig. 6:**
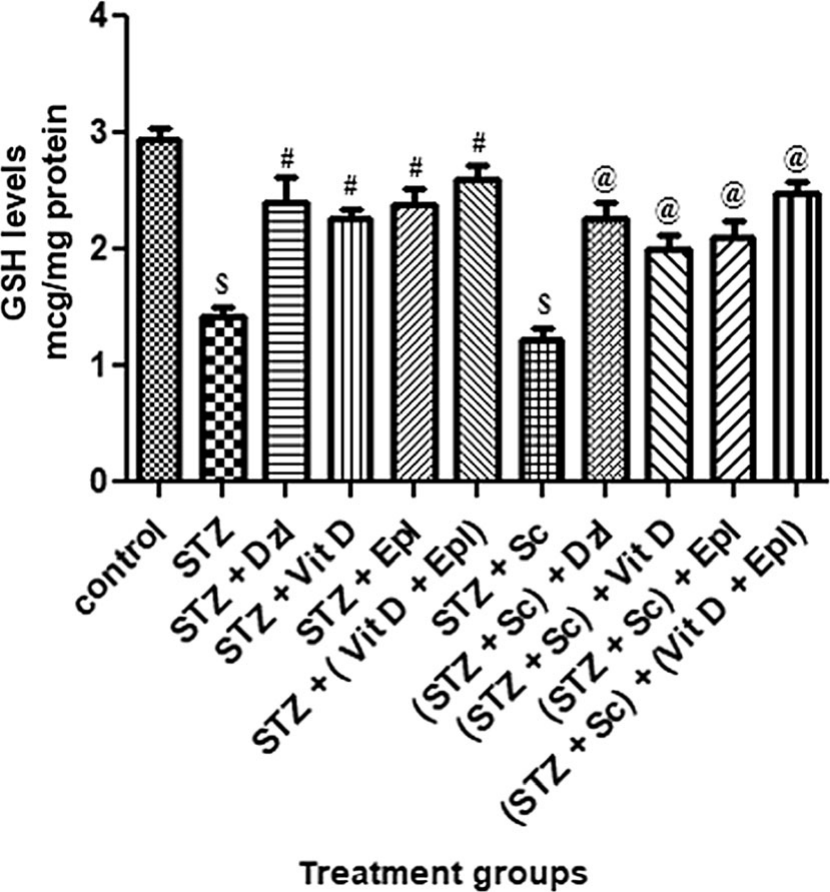
Effect of various drugs on GSH levels in brain homogenate. STZ: Streptozotocin, Sc: Scopolamine, Epl: Epalrestat, Vit D: Vitamin D, Dzl: Donepezil. ^$^*p* < 0.001 versus control, ^#^*p* < 0.05 versus STZ, ^@^*p* < 0.05 versus [STZ + Sc]. Values are expressed as Mean ± SD. Comparison is done by one way ANOVA followed by post hoc Tukey’s analysis

As depicted in Fig. 6, rats treated with STZ and (STZ + scopolamine) showed significant (*p* < 0.001) decrease in hippocampal GSH levels as compared to control rats. Among rats dosed with donepezil, vitamin D, epalrestat and (vitamin D + epalrestat) showed statistically significant (*p* < 0.05) elevation in GSH levels as compared to STZ and (STZ + scopolamine) group. However, the combination (vitamin D + epalrestat) treated group of rats statistically showed significant elevation in GSH levels in diabetic rats with and without the scopolamine; which were comparable with that of standard donepezil treated rats (Fig. 6).

#### Brain acetyl cholinesterase (AChE) estimation

**Fig. 7:**
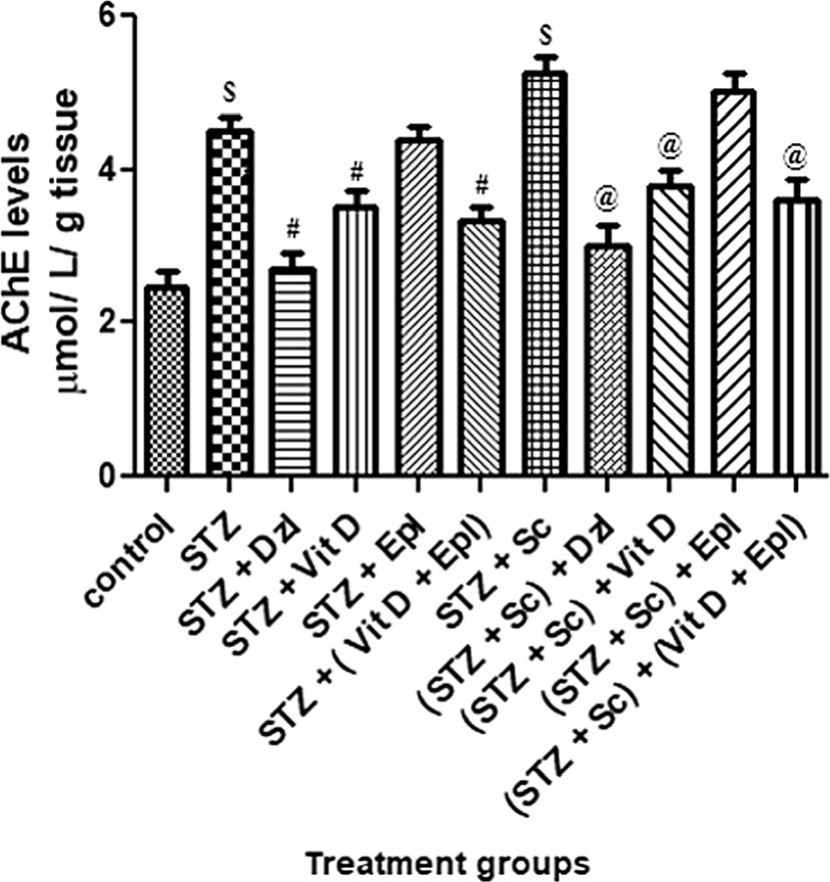
Effect of various drugs on AChE levels in brain homogenate. STZ: Streptozotocin, Sc: Scopolamine, Epl: Epalrestat, Vit D: Vitamin D, Dzl: Donepezil. ^$^*p* < 0.001 versus control, ^#^*p* < 0.05 versus STZ, ^@^*p* < 0.05 versus [STZ + Sc]. Values are expressed as Mean ± SD. Comparison is done by one-way ANOVA followed by post hoc Tukey’s analysis

As observed in Fig. 7, rats treated with STZ and (STZ + scopolamine) showed significant (*p* < 0.001) increase in brain AChE levels as compared to control rats. Rats treated with donepezil, vitamin D, and (vitamin D + epalrestat), AChE levels were significantly (*p* < 0.05) reduced as compared to STZ and (STZ + scopolamine) treated group of rats. However, the combination (vitamin D + epalrestat) treated group of rats statistically showed significant reduction in the AChE levels in diabetic rats with and without the scopolamine drug; which were comparable with that of donepezil treated rats (Fig. 7).

### Histopathological analysis

Histopathological changes were observed in CA1 of hippocampus under the microscope with 40X magnification. Hippocampal CA1 neurons (soma) with prominent nucleus, clear cytoplasm and healthy cell membrane were considered as normal neurons. On the other hand, flame shaped hippocampal CA1 neurons (soma) with pyknotic cell bodies (karyopyknosis), homogenous cytoplasm and intense basophilic appearance were considered as damaged or degenerated cells.

**Fig. 8:**
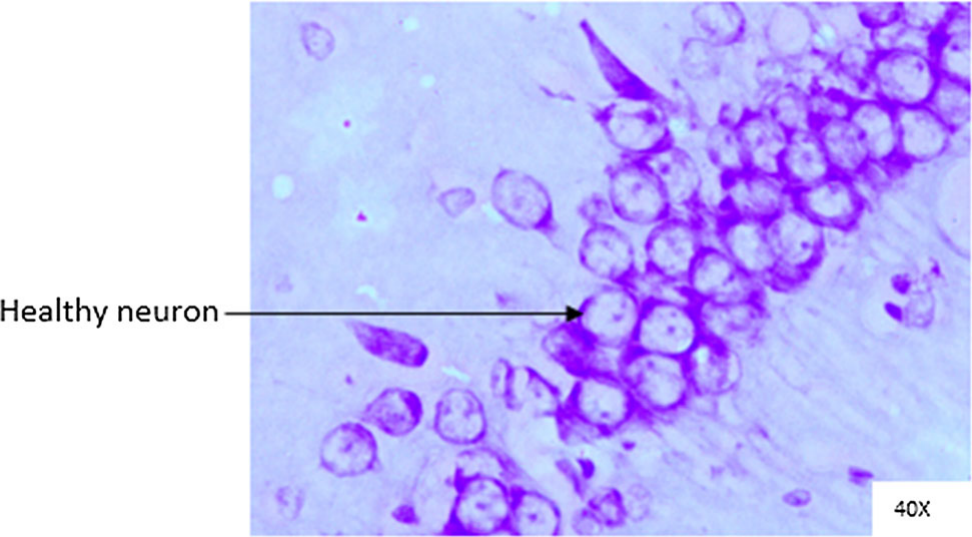
Histopathology of hippocampal CA1 region of group 1—control

**Fig. 9:**
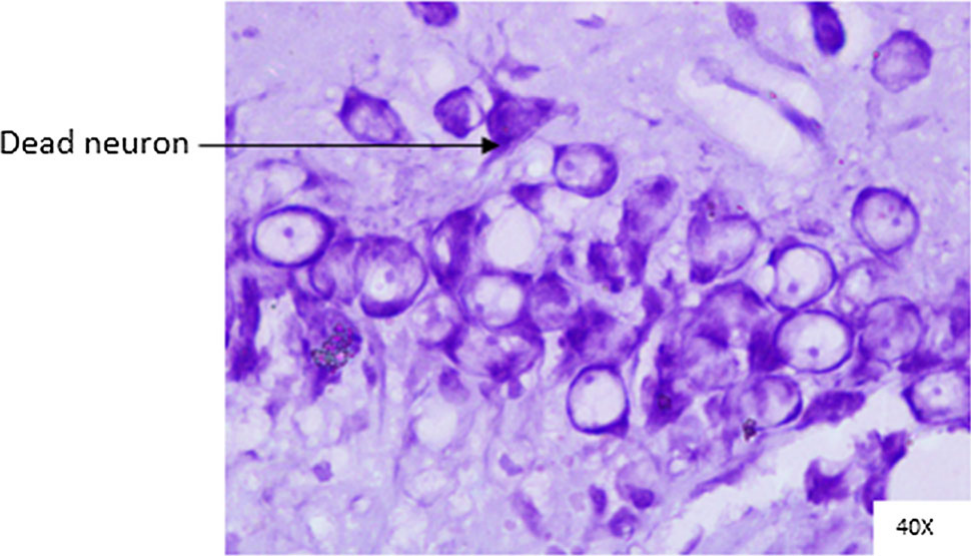
Histopathology of hippocampal CA1 region of group 2—STZ

**Fig. 10:**
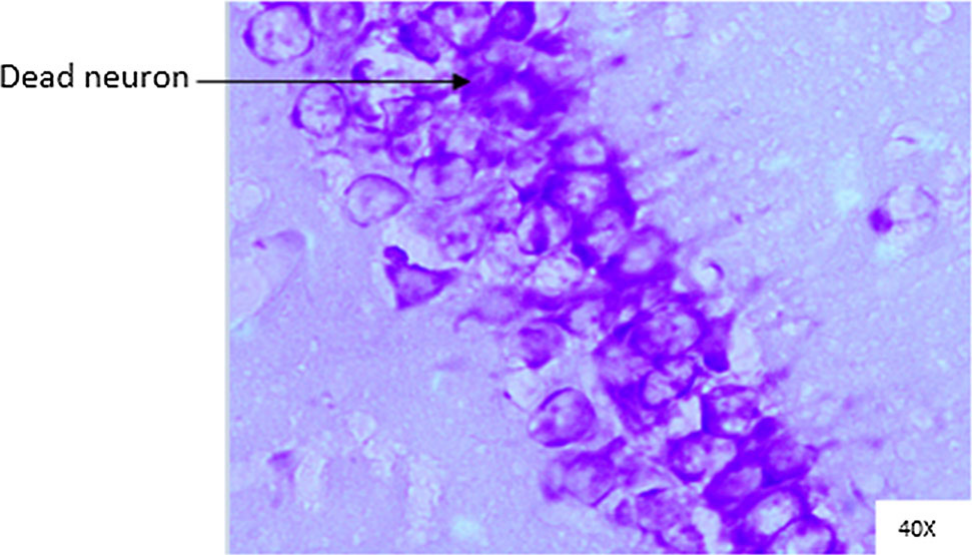
Histopathology of hippocampal CA1 region of group 7—STZ + scopolamine

**Fig. 11:**
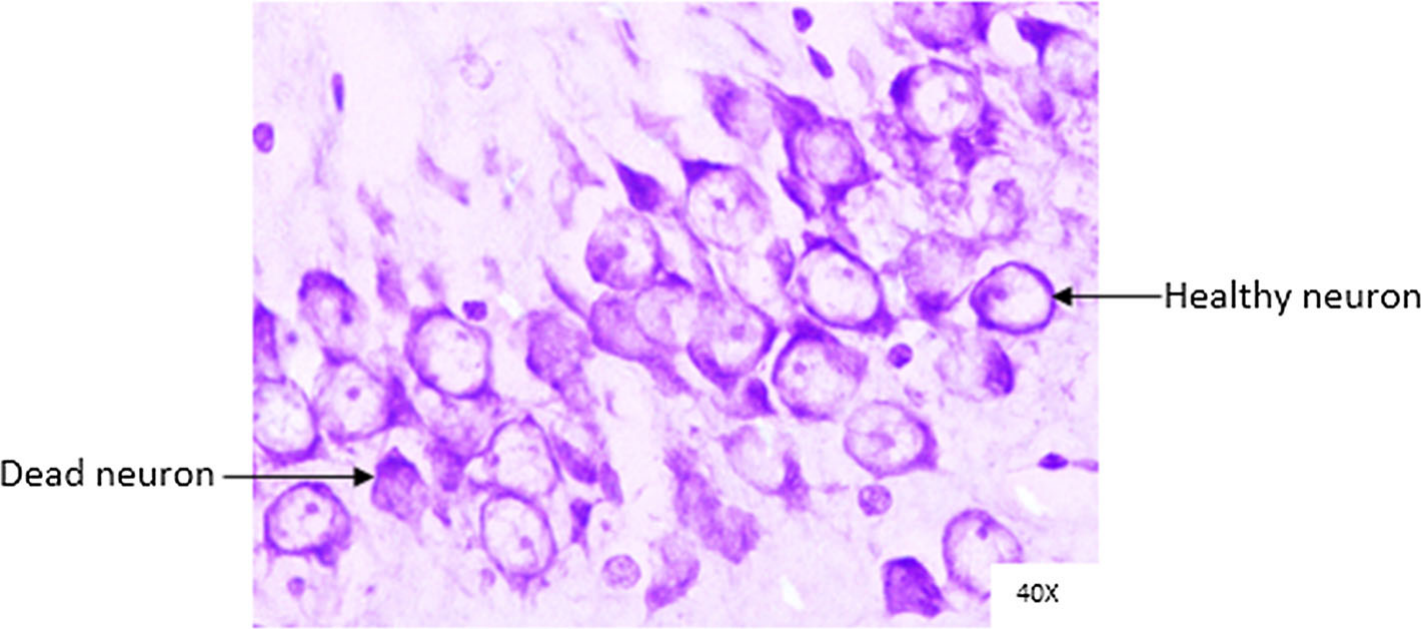
Histopathology of hippocampal CA1 region of group 4—STZ + vitamin D

**Fig. 12:**
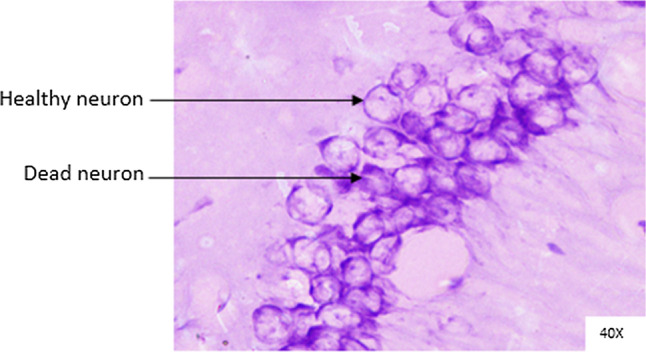
Histopathology of hippocampal CA1 region of group 9—(STZ + scopolamine) + vitamin D

**Fig. 13:**
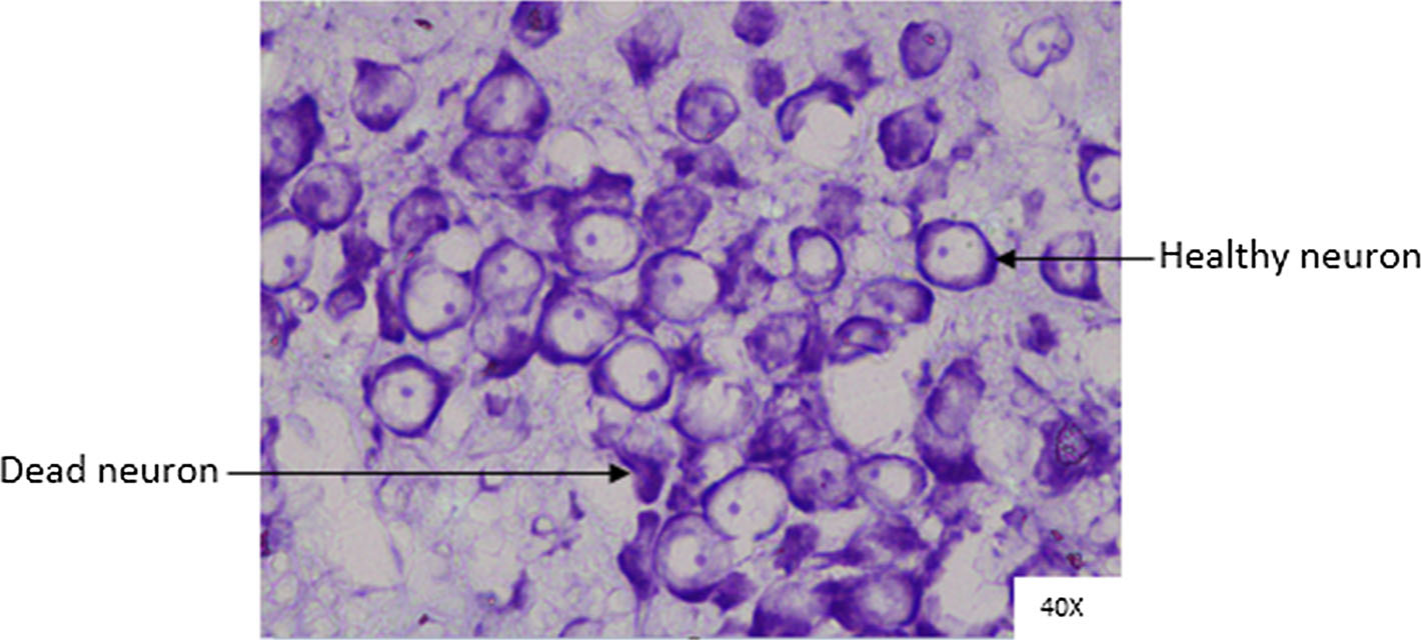
Histopathology of hippocampal CA1 region of group 5—STZ + epalrestat

**Fig. 14:**
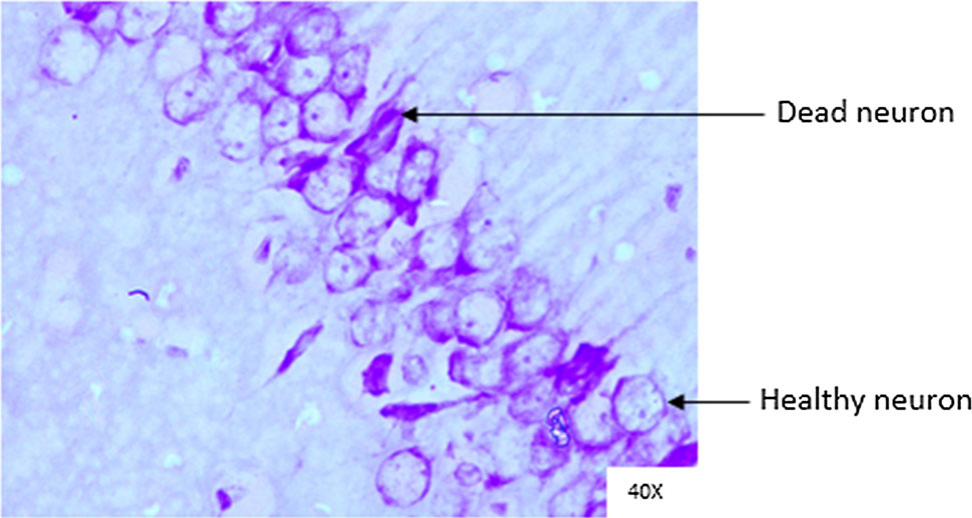
Histopathology of hippocampal CA1 region of group 10—(STZ + scopolamine) + epalrestat

In control rats (Fig. 8), no degenerative features were seen. Most of the neurons present in CA1 region were healthy with pale, round, well defined and prominent nuclei. In groups treated with STZ (Fig. 9) and (STZ + scopolamine) (Fig. 10), the sections showed many damaged neurons in CA1 region which were darkly (basophilic) stained, with shrunken and fragmented nuclei. Vacuoles were seen in hippocampal cells. Moderate to severe degenerative changes were observed. Brain section of groups treated with vitamin D (Figs. 11, 12) and epalrestat (Figs. 13, 14) a showed notably decreased damage of neurons.

**Fig. 15:**
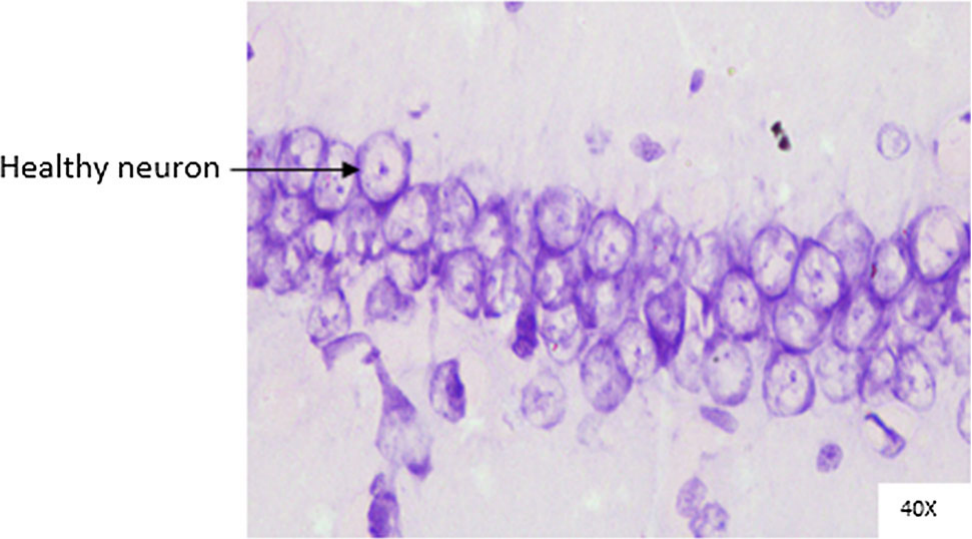
Histopathology of hippocampal CA1 region of group 3—STZ + donepezil

**Fig. 16:**
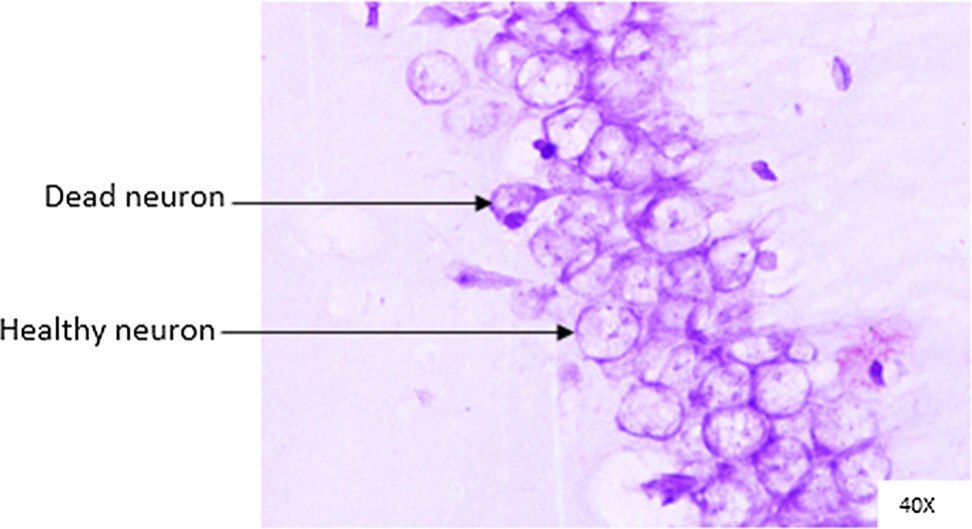
Histopathology of hippocampal CA1 region of group 8—(STZ + scopolamine) + donepezil

**Fig. 17:**
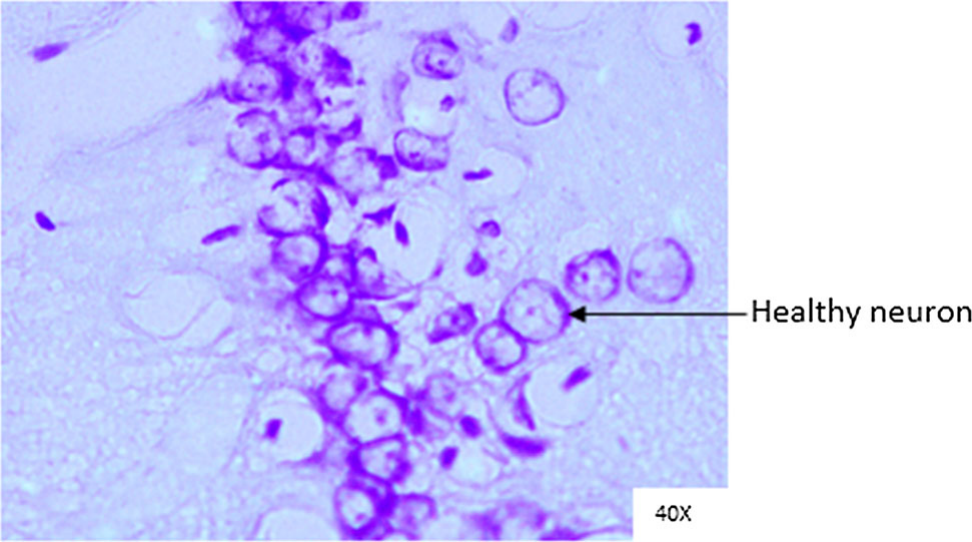
Histopathology of hippocampal CA1 region of group 6—STZ + (vitamin D + epalrestat)

**Fig. 18:**
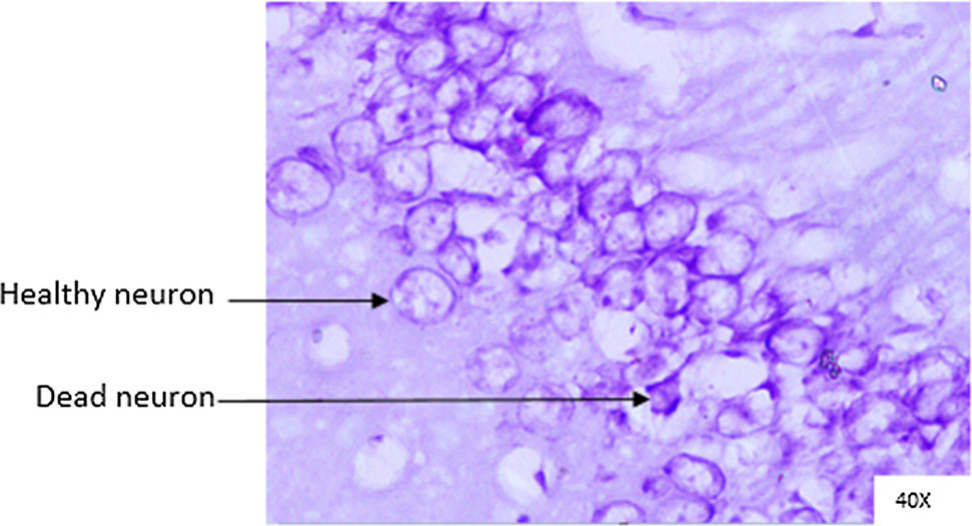
Histopathology of hippocampal CA1 region of group 11—(STZ + scopolamine) + (vitamin D + epalrestat)

Histopathological changes of hippocampus region of diabetic rats treated by donepezil; with and without scopolamine (Figs. 15, 16) and diabetic rats treated by (vitamin D + epalrestat) combination; with and without scopolamine (Figs. 17, 18) showed neuro-protective action with minimal neuronal damage.

## Discussion

The present study was conducted to screen and determine the neuroprotective, neuro modulatory and anti-oxidant activities of Vitamin D, epalrestat and its combination against persistent hyperglycaemia; with and without scopolamine induced amnesia in rodents. In humans, it is a known fact that diabetes status, oxidative stress, ageing and cholinergic nerve degeneration; triggering unique pathogenic pathways and mechanisms which are interlaced and proven to be deterrent in maintaining sound cognitive functions. There is no reason why a prolific researcher should not be focused in exploring any active pharmacological moieties or repositioning the drug in preventing cognition ailments. We found that amnesia produced by (STZ + HFD) induced diabetic rats treated with and without scopolamine was reversed by donepezil, Vitamin D, epalrestat and (Vitamin D + epalrestat) combination. Streptozotocin induced destruction of pancreatic beta cells and HFD induced obesity synergistically produce persistent hyperglycaemia. This uncontrolled hyperglycaemia has been related to lowered verbal memory, processing speed, episodic memory, instant recall, delayed recall, executive function, visual perception, and responsiveness (Kodl and Seaquist 2008). Bruce et al. (2003) found that 17.5% of elderly patients with type 2 diabetes had moderate to severe deficits in activities of daily living, 11.3% had cognitive impairment, and 14.2% had depression. Multiple reports have shown an inverse association between blood sugar levels and working memory, learning, and complex neurocognitive output in type 2 diabetic patients, encouraging the hypothesis that deteriorating glucose regulation contributes to declining cognitive performance (Kodl and Seaquist 2008). Diabetic rats were also treated separately with scopolamine, which is one of the gold standard models for inducing cognitive decline and memory difficulties. The antiamnesic activity of test drugs was compared to donepezil.

In the current study, type-2 diabetic rats exhibited marked impairment in learning and memory that was revealed with various behavioural parameters in MWM tasks and PA test. MWM is a hippocampus-dependent memory task that is commonly used in rodents to show cognitive deficiencies in permanent spatial learning ability and reference memory (Vittalrao et al. 2019). The PA model is the most frequently used fear-aggravated test for screening potential cognition enhancers in central nervous system disorders, with reproducible and dependable results (Vittalrao et al. 2019). In the probe test session of MWM, percentage of time spent and percentage of distance travelled in target quadrant by groups treated with donepezil, vitamin D, epalrestat and (vitamin D + epalrestat) was markedly higher in comparison with diabetic and (diabetic + scopolamine) groups of rats. However, it was maximum in donepezil followed by (vitamin D + epalrestat) treated rats. This justifies that the rats treated with donepezil and (vitamin D + epalrestat) displayed an improvement in the intensity and precision of recalling the previous navigation memory. Common mechanisms used for "visuospatial navigation" in rats also relate significantly to day-to-day human cognitive functions (Terry 2009). The PA model, rats showed deleterious behavioural effects of diabetic and (diabetic + scopolamine) treatment by a decrease in the avoidance latency compared to control rats. However, rats given donepezil, vitamin D, epalrestat, and a combination of the two (vitamin D + epalrestat) were able to successfully learn that the dark compartment was electrified, resulting in a foot shock, as evidenced by a significant increase in the time it took to enter the dark compartment or step through latency (STL). Hence the latency time to enter the dark compartment was notably increased and percentage of time spent was decreased. This suggests that rats treated with (vitamin D + epalrestat) and standard drug donepezil facilitated PA responding by increasing the latency to enter the dark compartment and also prevented cognitive impairment caused by chronic hyperglycaemia with and without scopolamine. The present results support the notion that chronic diabetic state and scopolamine impaired the learning of the inhibitory response primarily by acting on the consolidation and retention phases of memory formation. Our test drug (vitamin D + epalrestat) and standard drug donepezil prevented memory impairment in both training and retention phases, most probably by alleviating oxidative stress and facilitating cholinergic transmission respectively thus improving the learning and memory processes in rats. These results concord that cognitive impairment in STZ-induced diabetic rats, is associated with hippocampal dysfunction.

Another well-known causative element in the pathophysiology of dementia and age-related neurodegenerative illnesses, such as Alzheimer's disease, is oxidative stress. Because of its high oxygen consumption (20%), metal concentration, polyunsaturated fatty acids, and limited anti-oxidant capacity, brain tissue is particularly sensitive to oxidative stress. The brain contains only low to moderate levels of antioxidant enzymes, such as catalase, SOD (superoxide dismutase), and GPx (glutathione peroxidase), compared with other organs, and GSH is the most prevalent anti-oxidant in most brain cells (Hafez et al. 2017). Hyperglycaemia increases flux through the polyol pathway in nervous tissue. In the study conducted by *Kodl CT*. STZ-treated hyperglycaemic rats, showed increased sorbitol in cranial nerves, sciatic nerve, cerebral cortex, and retina (Kodl and Seaquist 2008). Increased intracellular sorbitol in neurons causes tremendous increase in oxidative stress and release of reactive oxygen species (ROS). Greater oxidative injury was seen in the brains of experimentally induced hyperglycemic rats. Oxidative stress induces inflammation and, consequently, raises inflammatory mediators. Inflammation is regarded as an effective pathophysiological phenomenon in type 2 diabetes which could possess a part to perform in the vulnerability of type 2 diabetes patients to neurodegenerative diseases (Jaiswal et al. 2018b). Glutathione (GSH), the most important antioxidant, has a vital activity in maintaining the cellular redox state. Thalamus and hypothalamus are more susceptible to free radical damage as evidenced by the decrease in GSH levels in these regions. Hippocampus is mainly responsible for the collection, consolidation and retrieval of various aspects of learning, memory and spatial navigation (Jaiswal et al. 2018b). Elevated hippocampal oxidative stress corresponding to reduced content of glutathione and raised levels of MDA are considered as reliable indices of oxidative stress and our study showed deranged cognition most probably due to the oxidative damages observed in hippocampus.

In the current study, rats given donepezil, vitamin D, epalrestat, or the combination (vitamin D + epalrestat) significantly improved memory deficits caused by diabetic rats with and without scopolamine, as well as significantly alleviating oxidative stress by lowering MDA and increasing GSH-level activities; which corresponds to the results of previous studies conducted by *Jaiswal S, and Kodl CT* (Kodl and Seaquist 2008; Jaiswal et al. 2018a). Other, neurochemical changes, including decreased acetylcholine have been observed (Kodl and Seaquist 2008). The modulating AChE activity could affect the underlying neuronal processes which encouraged us to measure AChE levels. The pretreatment with donepezil, vitamin D, epalrestat, or the combination (vitamin D + epalrestat) significantly reduced the AChE activity and the loss of cholinergic activity in the hippocampus as compared with the diabetic rats with and without scopolamine. The inhibitory activity of (vitamin D + epalrestat) comparable to standard drug donepezil. The diabetic rats with and without scopolamine possibly inflicted severe deficits in cholinergic neuron functionality, augmented AChE activity and expression in the hippocampus enhancing the neurodegeneration in the brain. The possible neuro-modulatory mechanism by test donepezil and (vitamin D + epalrestat) drugs could be due to inhibition of AChE activity, increasing the release of ACh and prolonging the duration of acetylcholine in the brain. These finding could be attributed to central cholinergic neuro-modulatory properties of vitamin D similar to donepezil (cerebro-selective anti-cholinesterase) (Alrefaie and Moustafa 2020).

These findings corroborate to the fact that the test drugs (vitamin D + epalrestat) and donepezil treated group of rats showed significant neuro-protective activities against diabetic rats treated with and without scopolamine; probably by modulating neuro inflammatory processes, learning memory processes, anti-oxidant, and maintaining neuronal structural integrity.

Vitamin D is a steroid hormone that plays an important role in the central nervous system and has neuroprotective properties. There is enough cumulative evidence suggesting vitamin D receptors and important enzymes involved in its metabolism are scattered throughout the brain of rats, particularly in areas afflicted by neurodegenerative illnesses. Vitamin D has been proposed to cross through the BBB and have a direct effect on the central nervous system. Animal studies have shown that the function of the hippocampus, a pivotal region of the brain involved in cognition, is adversely affected by obesity. Neuronal hippocampal inflammation, neurotrophin decrease, and alteration of cerebral vascular function, such as BBB integrity in HFD fed mice, are some of the postulated underlying processes. Our study results were partly in agreement with G Hajiluian et al. who reported that Vitamin D significantly increased time spent in MWM probe test, oxidative stress and reversed HFD-induced cognitive impairments via reduction of NF-κB concentrations, BBB permeability and elevation of BDNF (Brain-derived neurotrophic factor) in the hippocampus (Hajiluian et al. 2017).

Diabetic neuropathy is caused by an increase in the activity of aldose reductase, a major enzyme in the polyol pathway. Epalrestat (EPS) is a reversible aldose reductase inhibitor that lowers intracellular sorbitol, which is thought to be the aetiology of diabetic neuropathy. However, it has no effect on glycaemic control. The improvement in sorbitol levels and Na^+^/K^+^ ATPase activity that leads to better nerve conduction velocity has been demonstrated in animal tests (Ramirez and Borja 2008; Steele et al. 1993).

The anti-oxidant, neuro modulatory and nerve cell protective role of epalrestat underscored by the recent studies; heme oxygenase (HO)-1 possesses significant antioxidant and anti-inflammatory activities, according to earlier results; nevertheless, epalrestat activates Nrf2 (Nuclear factor erythroid 2-related factor 2) and upregulates HO-1, dismutase, and catalase, implying that it has a favourable influence on the improvement of numerous neurological illnesses (Yama et al. 2016). Epalrestat lowers intracellular sorbitol accumulation in hyperglycemic situations, which has been linked to the pathophysiology of diabetic complications (Steele et al. 1993). Epalrestat is quickly absorbed by brain tissue and effectively inhibits aldose reductase with few side effects. A recent study found that treating diabetic neuropathy with EPS at an early stage avoided the onset/progression of retinopathy and nephropathy (Hotta et al. 2012). Recently, we found that EPS at near-plasma concentration increases the intracellular levels of glutathione (GSH) in rat Schwann cells. GSH, the most abundant non-protein thiol antioxidant in cells, is important for protection against oxidative stress (Yama et al. 2015).

On histopathological analysis, it was perceived that in STZ and (STZ + scopolamine) group, neuronal cells suffered from inflammatory damage due to increased oxidative stress caused by diabetes, leaving behind ruptured flame shaped cells with no nuclei and disintegrated cell membrane. Due to scopolamine injection, the damage was strikingly more with maximum number of apoptotic dead cells. In groups treated with vitamin D and epalrestat, reasonable number of neurons were preserved despite the damage due to inflammation. The (vitamin D + epalrestat) offered better protection against inflammatory damage and managed to preserve significantly more number of neurons. Donepezil being a standard drug managed to keep the oxidative stress in check. These findings support the fact that the test drugs (vitamin D + epalrestat) and donepezil-treated rats showed significant neuro-protective activity against diabetic rats treated with and without scopolamine, possibly by modulating neuro-inflammatory processes, anti-oxidant activity, and maintaining neuronal structural integrity.

## Conclusion

The present study demonstrated that vitamin D and epalrestat, individually as well as in combination possess cognition enhancing ability against scopolamine induced amnesia in diabetic rat model by inhibiting lipid peroxidation and augmenting endangered antioxidants. In addition to this, vitamin D also exhibited reduction in acetylcholinesterase activity in brain which was also displayed by the combination of vitamin D and epalrestat. Ultimately, our study indicated that cognition enhancing ability of combination of vitamin D and epalrestat is comparable with that of the standard drug donepezil. However, further studies are warranted to detect the potential use of the combination of vitamin D and epalrestat for cognitive enhancement in humans.

## Data Availability

On request will be shared.
